# Robot-Assisted Radical Prostatectomy as the Institutional Standard: Complete Transition and Contemporary Outcomes from a High-Volume European Center

**DOI:** 10.3390/jcm15124606

**Published:** 2026-06-13

**Authors:** Simon Hawlina, Andraž Kondža, Kosta Cerović, Jure Bizjak

**Affiliations:** 1 Department of Urology, University Medical Centre Ljubljana, 1000 Ljubljana, Slovenia; simon.hawlina@kclj.si (S.H.); andraz.kondza@kclj.si (A.K.);; 2 Faculty of Medicine, University of Ljubljana, 1000 Ljubljana, Slovenia

**Keywords:** robot-assisted radical prostatectomy, prostate cancer, robotic surgery, surgical outcomes, minimally invasive surgery, perioperative outcomes, healthcare transformation

## Abstract

**Background**: Robot-assisted radical prostatectomy (RARP) is the predominant surgical approach for localized prostate cancer in high-volume centers worldwide. However, comprehensive real-world data describing complete institutional transition from open to robotic surgery remain limited. This study evaluated perioperative and early oncological outcomes of a contemporary RARP cohort and characterized the transition from open radical prostatectomy (ORP) to RARP in a European center. **Methods**: We analyzed 520 consecutive patients who underwent RARP between January 2023 and December 2025. Perioperative, pathological, and biochemical outcomes were assessed. Biochemical recurrence was defined as prostate-specific antigen ≥0.2 ng/mL. Institutional data from 2011 to 2025 were reviewed to evaluate procedural trends and the transition from ORP to RARP. Surgeon-specific and institutional learning curves were analyzed using operative time and linear regression models. **Results**: Following the introduction of robotic surgery in 2018, annual RARP volume increased from 37 procedures to 205 in 2025. Since 2023, RARP accounted for more than 99% of all radical prostatectomies. Median operative time decreased from 185 min in 2023 to 165 min in 2025, with consistent downward trends observed across all surgeons. Linear regression confirmed progressive improvement in operative efficiency, with learning rates ranging from −0.22 to −0.92 min per case. Estimated blood loss was minimal, no patients required transfusion, and major complications occurred in four patients (0.8%). Hospital stay decreased from 2 days to predominantly 1 day. During follow-up, 36 patients developed biochemical recurrence or PSA persistence. Biochemical recurrence-free survival differed significantly according to pathological stage (log-rank *p* < 0.001), with 24-month estimates of 93.7%, 91.5%, and 82.1% for pT2, pT3a, and pT3b disease, respectively. **Conclusions**: RARP provides favorable perioperative safety, minimal morbidity, and favorable early oncological outcomes in a high-volume setting. The complete institutional transition from ORP to RARP, together with demonstrated surgeon-specific and institutional learning effects, supports the feasibility and safety of implementing RARP as the institutional standard within a structured robotic program.

## 1. Introduction

Radical prostatectomy is the cornerstone in the management of localized prostate cancer. Over the past two decades, surgical techniques have evolved from open radical prostatectomy (ORP) to minimally invasive approaches, including laparoscopic and robot-assisted radical prostatectomy (RARP) [1,2]. In contemporary high-volume centers and national cohorts, RARP has become the most commonly performed approach for radical prostatectomy worldwide [2,3]. The adoption of robotic surgery has been driven by improved visualization, enhanced dexterity, and the potential for reduced blood loss, shorter hospital stay, and faster postoperative recovery [4,5,6]. Several studies have demonstrated comparable or improved perioperative and early oncologic outcomes with RARP compared with ORP [4,7,8]. In addition to improved surgical ergonomics and visualization, robot-assisted surgery has been increasingly associated with lower blood loss, shorter hospitalization, and reduced perioperative morbidity, including lower complication rates and faster postoperative recovery [5,7,9]. Understanding how these benefits translate at the institutional level is essential for optimizing surgical care pathways.

Despite widespread adoption, the process of transitioning from an open to a fully robotic surgical program at the institutional level has been insufficiently described. Most available studies focus on early adoption phases or learning curves for individual surgeons. Comprehensive analyses of complete institutional transformation are limited [10,11,12,13].

In Slovenia, the introduction of robotic surgery at the University Medical Centre Ljubljana led to a rapid and complete shift in prostate cancer surgery. Over a relatively short period, the institution transitioned from predominantly open procedures to an almost exclusively robotic approach.

The aim of this study was to evaluate perioperative and early oncologic outcomes in a contemporary real-world cohort of patients undergoing RARP between 2023 and 2025 and to contextualize these findings within the complete institutional transition from ORP to RARP between 2011 and 2025. To our knowledge, this is one of the few studies describing a complete institutional transition to robotic prostatectomy in a European center.

## 2. Materials and Methods

### 2.1. Study Design and Patient Population

This retrospective single-center study included consecutive patients who underwent RARP at the University Medical Centre Ljubljana between January 2023 and December 2025. Follow-up data were updated through to April 2026. Historical institutional data from 2011 to 2025 were also reviewed to characterize procedural trends and the transition from ORP to RARP. Only primary robot-assisted radical prostatectomies performed for localized prostate cancer were included. Salvage prostatectomy procedures were not included in the study cohort. No additional exclusion criteria were applied.

Although robotic surgery was introduced at our institution in 2018, the primary outcomes analysis was restricted to patients treated between 2023 and 2025. This period was selected because a standardized prospective institutional database was implemented in 2023, enabling comprehensive and reliable collection of perioperative, pathological, complication, and oncological outcomes. While selected procedural data were available from earlier years, these records were incomplete for several key variables and were therefore considered unsuitable for a detailed contemporary outcomes analysis. Earlier institutional data were consequently used to illustrate the transition from open to robotic surgery, whereas the 2023–2025 cohort served as the basis for outcome analyses.

Robotic prostatectomy was introduced at our institution in 2018. Prior to January 2023, 485 robot-assisted radical prostatectomies had been performed. Surgeon 3 initiated the robotic program in June 2018 and performed the majority of procedures during the implementation phase. Surgeon 1 joined the program in September 2018 and Surgeon 4 in January 2021, each subsequently contributing substantially to institutional case volume. Surgeon 2 started robotic training in February 2023 and had no prior independent robotic prostatectomy experience at study entry.

During surgeon onboarding, procedures were performed under the supervision of experienced robotic surgeons. Surgeon 3 served as the primary mentor for Surgeons 1, 2, and 4. In addition, Surgeons 1 and 4 participated in the training of Surgeon 2. The institutional robotic program was initially established with the support of an experienced external proctor during the first four robotic cases. This structured mentorship model should be considered when interpreting the surgeon-specific learning curve analyses presented in this study.

Baseline patient and tumor characteristics are summarized in Table 1.

### 2.2. Surgical Technique

All procedures were performed using the da Vinci Xi robotic system (Intuitive Surgical, Sunnyvale, CA, USA) by three fully trained and experienced robotic surgeons and one surgeon in the early phase of the robotic learning curve, using a standardized transperitoneal robot-assisted approach within a structured institutional program.

**Figure 1:**
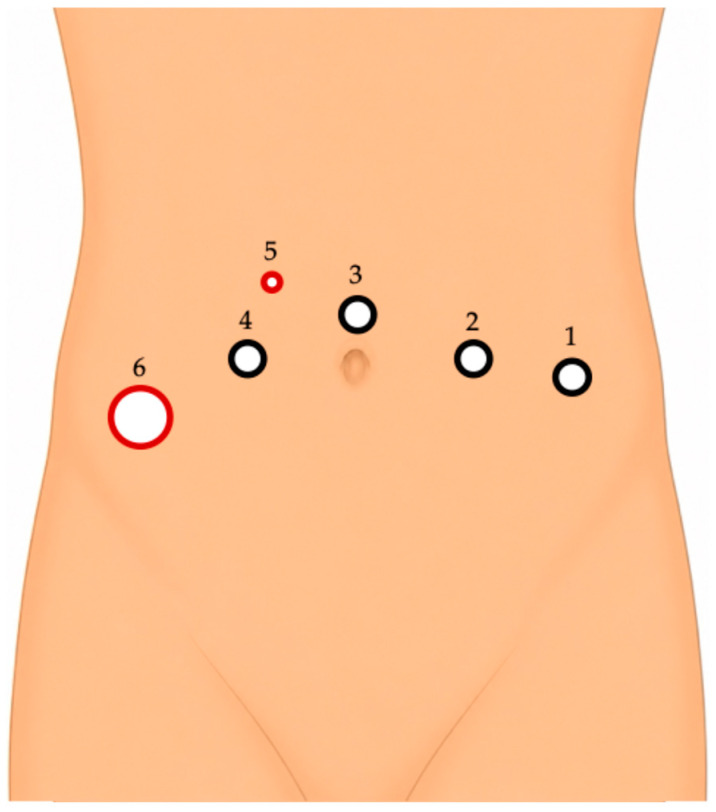
Standardized trocar placement for robot-assisted radical prostatectomy using the da Vinci Xi system (1, 2, 4—8 mm robotic working ports, 3—camera, 5—5 mm assistant port, 6—AirSeal^®^ port).

Trocar placement is standardized: the camera port is positioned slightly above the umbilicus, with three robotic working ports placed approximately 2 cm inferiorly on the right and left sides (Figure 1). A 5 mm assistant port is positioned between the camera and the right robotic arm, and an AirSeal^®^ port is placed in the far right lateral position to ensure stable pneumoperitoneum and efficient smoke evacuation. The procedure is performed using low-pressure pneumoperitoneum (8 mmHg), which contributes to reduced physiological impact and aligns with the principles of low-impact robotic surgery, as previously described in the literature [14].

A modified anterior “hood” technique, as described by Mandel et al. [15], was systematically applied. This approach emphasizes maximal preservation of periurethral anatomical structures, including the detrusor apron and anterior support mechanisms, with the aim of minimizing urethral mobility and improving early postoperative continence. After establishing pneumoperitoneum and trocar placement, the procedure proceeds in a controlled and standardized manner. The operative field is prepared with clear exposure of anatomical landmarks. The bladder neck is carefully identified and opened using a single, precise anterior incision, allowing for controlled entry into the bladder while preserving surrounding supportive structures to the greatest extent possible. This is followed by dissection and isolation of the seminal vesicles (vesiculae seminales) and vas deferens (ductus deferentes), which are subsequently transected. Posterior dissection is performed with attention to anatomical planes and preservation of surrounding structures.

Whenever oncologically appropriate, nerve-sparing surgery was performed. Preservation of the neurovascular bundles was graded according to the Tewari classification, where Grade 1 represents maximal nerve preservation and Grade 4 represents a non–nerve-sparing dissection [16]. A strict “no-clip” technique is employed throughout the procedure, with hemostasis achieved using precise energy application (pinpoint monopolar coagulation), thereby minimizing tissue trauma and avoiding foreign material.

Anterior dissection of the prostate is then completed while preserving the dorsal venous complex (DVC). The urethra is clearly visualized and transected at the level of the prostatic apex, allowing for complete mobilization of the specimen, which is subsequently placed into an endoscopic retrieval bag. Reconstruction is performed in a standardized fashion. Posterior reconstruction (Rocco stitch) is carried out to restore anatomical support, followed by a watertight vesicourethral anastomosis. Additional reconstruction of the detrusor apron is performed to further enhance periurethral support. The specimen is extracted through a small assistant incision in the right lower abdominal quadrant.

Pelvic lymph node dissection (PLND) was performed selectively according to preoperative oncological risk stratification and surgeon judgement. In general, PLND was considered in patients with intermediate- and high-risk disease or when the estimated risk of lymph node involvement exceeded 7%, in accordance with contemporary guideline recommendations. The final decision was individualized based on patient and disease characteristics. Whenever lymphadenectomy was performed, an extended pelvic lymph node dissection (ePLND) template was used. Nodal status was available only in patients undergoing ePLND. Patients who did not undergo lymph node dissection were classified as pNx.

Estimated blood loss was recorded intraoperatively by the anesthesia team based on the volume collected in the suction canister and documented in the operative record.

Perioperative management followed enhanced recovery protocols, contributing to early mobilization and reduced length of hospital stay.

Postoperative follow-up was performed according to the institutional protocol. Cystography was routinely performed approximately 7 days after surgery, and the urinary catheter was removed if no evidence of vesicourethral leakage was observed. The first postoperative PSA measurement was obtained approximately 6 weeks after surgery, followed by routine clinical assessment at 3 months. Subsequent follow-up was performed according to institutional practice and oncological risk assessment.

### 2.3. Data Collection

Clinical and perioperative data were collected from institutional databases and electronic medical records. The following variables were analyzed: operative time, estimated blood loss, length of hospital stay, perioperative complications (Clavien–Dindo classification), pathological outcomes, and biochemical outcomes.

Data completeness was assessed for all study variables. Analyses were performed using available-case methodology, and denominators are reported for variables with incomplete data. No imputation of missing values was performed.

### 2.4. Outcome Measures

The primary endpoints were perioperative outcomes and early oncologic results. Biochemical recurrence (BCR) was defined as a prostate-specific antigen (PSA) level ≥0.2 ng/mL [17]. Patients with PSA persistence, defined as failure of PSA to decline below the lower limit of assay detection following radical prostatectomy, were considered to have experienced an oncological event and were included in the biochemical recurrence analysis. BCR-free survival was estimated based on observed follow-up.

### 2.5. Statistical Analysis

Descriptive statistics were used to summarize outcomes. Continuous variables are presented as medians (interquartile range), and categorical variables as frequencies and percentages. Temporal trends were assessed descriptively to evaluate changes in operative efficiency over time.

Surgeon-specific and institutional trends in operative efficiency were assessed using operative time as a surrogate marker of operative efficiency. For each surgeon, case sequence was used as the independent variable and operative time as the dependent variable. Linear regression models were fitted separately for each surgeon to estimate changes in operative time over consecutive cases.

In addition, an institutional trend was generated to evaluate overall changes in operative efficiency across the robotic program. Extreme outliers in operative time (>400 min) were excluded from the learning-curve visualization to improve interpretability, but the overall perioperative outcome analysis was based on the full available dataset.

## 3. Results

### 3.1. Institutional Transition and Surgical Volume

Perioperative outcomes are presented in Table 2.

Major complications (Clavien–Dindo ≥ IIIa) occurred in four patients (0.8%). These included two postoperative bleeding events, one postoperative ileus, and one thromboembolic event. Two patients required reoperation, including one patient with postoperative hematoma and one patient with postoperative ileus. No lymphoceles requiring drainage, urinary leaks requiring intervention, or blood transfusions were observed.

Additional surgical characteristics are summarized in Table 2. Full nerve-sparing surgery (bilateral Tewari Grade 1) was achieved in 332 patients (67.5%), while a non-nerve-sparing approach was required in only 2 patients (0.4%). Extended pelvic lymph node dissection (ePLND) was performed in 59 patients (11.3%) according to preoperative risk assessment, with a median lymph node yield of 10 (IQR 5–14).

**Figure 2:**
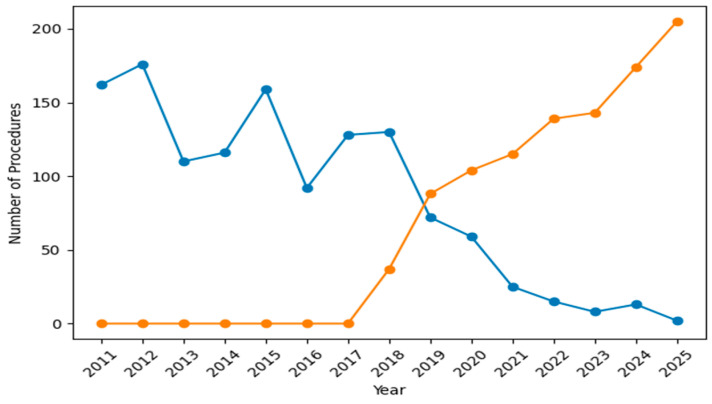
Institutional transition from open to robot-assisted radical prostatectomy (2011–2025). Annual numbers of open (ORP, blue line) and robot-assisted (RARP, orange line) procedures performed between 2011 and 2025.

There was a rapid and sustained increase in the adoption of RARP after its introduction in 2018. Annual volume increased from 37 procedures in 2018 to 205 in 2025, a more-than-fivefold increase (Figure 2). The increase to >200 RARP procedures annually places our institution among the high-volume European robotic prostatectomy centers [18].

Since 2023, robotic surgery has accounted for more than 99% of all radical prostatectomies, reflecting a complete institutional transition within approximately 7.5 years. The annual procedural volumes demonstrate the progressive replacement of ORP by RARP at our institution.

### 3.2. Perioperative Outcomes

Five hundred and twenty patients underwent RARP between 2023 and 2025. The median operative time decreased from 185 to 165 min, suggesting progressive optimization and increasing procedural efficiency over time.

Blood loss was minimal; no transfusions were required. Major complications occurred in four patients (0.8%), confirming the safety of the robotic approach.

Hospital stay decreased from predominantly 2 days to 1 day, reflecting both surgical refinement and optimized perioperative care pathways.

### 3.3. Surgeon-Specific and Institutional Learning Curve

Surgeon-specific analysis of operative time demonstrated a consistent downward trend across all four surgeons, indicating progressive improvement in operative efficiency over consecutive cases. Linear regression confirmed negative slopes for each surgeon, with estimated learning rates ranging from −0.22 to −0.92 min per case.

**Figure 3:**
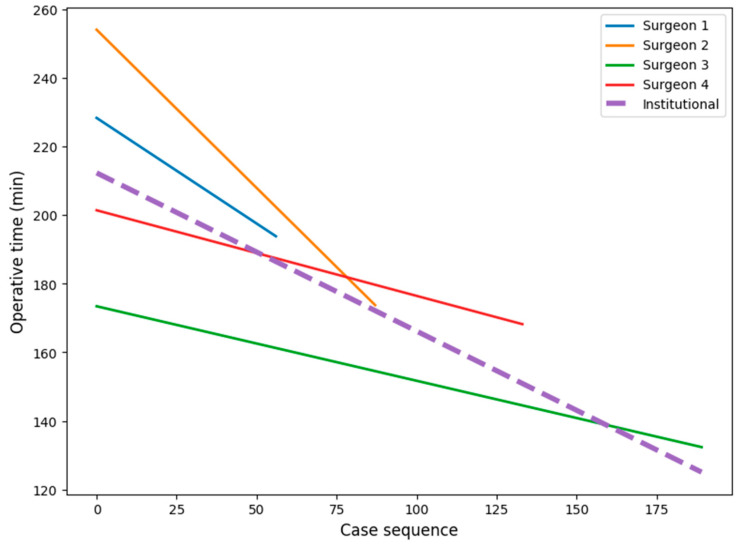
Surgeon-specific and institutional learning curves for operative time. Linear regression trend lines demonstrate progressive reduction in operative time across all surgeons, while the institutional trend line reflects overall improvement in procedural efficiency at the program level.

The most pronounced reduction in operative time was observed in surgeons with lower prior robotic experience, whereas surgeons with higher accumulated case volumes demonstrated flatter slopes, consistent with performance stabilization over time. Specifically, the estimated slopes were −0.62 min/case for Surgeon 1 (95% CI, −1.21 to −0.02), −0.92 min/case for Surgeon 2 (95% CI, −1.27 to −0.58), −0.22 min/case for Surgeon 3 (95% CI, −0.31 to −0.13), and −0.25 min/case for Surgeon 4 (95% CI, −0.40 to −0.10). At the institutional level, the aggregated trend line also demonstrated a clear reduction in operative time, suggesting improved operative efficiency over time at both the surgeon and institutional level (Figure 3).

Although variability in operative time was observed between surgeons, the overall trend showed convergence toward a similar operative time range in the later phase of the study period, reflecting increasing consistency of performance at the institutional level.

### 3.4. Pathologic Outcomes

Pathological outcomes are summarized in Table 3.

The majority of patients had organ-confined disease, with pT2 stage observed in 72.5% of cases, while locally advanced disease (pT3a and pT3b) was present in 27.5% of patients.

Extended pelvic lymph node dissection (ePLND) was performed in 59 patients (11.3%) according to preoperative risk assessment. Among patients undergoing ePLND, the median lymph node yield was 10 (IQR 5–14). Lymph node metastases were identified in 16 patients, whereas 43 patients were node-negative on pathological examination. Patients who did not undergo ePLND were classified as pNx.

Negative surgical margins (R0) were observed in 73.2% of patients, whereas positive surgical margins (R1) were identified in 26.8% of cases. Positive surgical margins were more frequent in locally advanced disease. The R1 rate was 19.4% in pT2 disease, increasing to 41.0% in pT3a disease and 52.9% in pT3b disease.

During follow-up, 36 patients developed biochemical recurrence or PSA persistence. Biochemical recurrence occurred in 11 of 344 patients (3.2%) with negative surgical margins and in 25 of 126 patients (19.8%) with positive surgical margins (Table 3). Positive surgical margins were associated with a more-than-six-fold higher rate of biochemical recurrence or PSA persistence (19.8% vs. 3.2%).

### 3.5. Early Oncologic Outcomes

**Figure 4:**
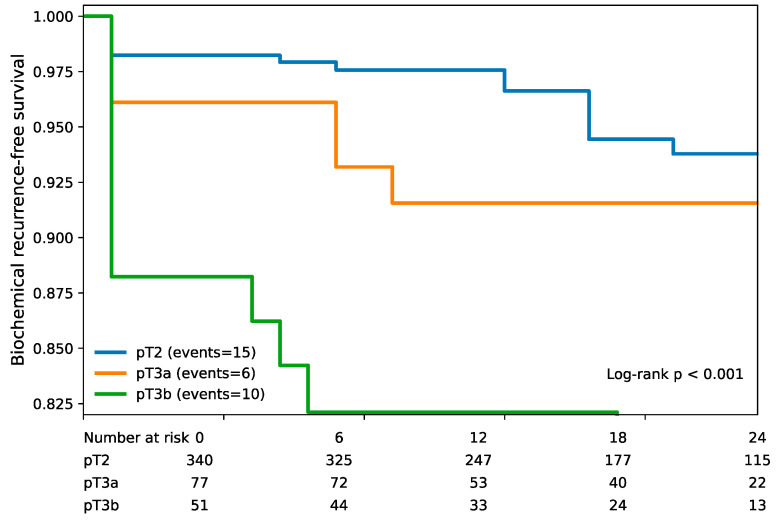
Kaplan–Meier estimates of biochemical recurrence-free survival according to pathological T stage. Biochemical recurrence included PSA persistence. The figure shows the number of patients at risk and the number of events in each subgroup; curves were truncated at 24 months.

During follow-up, 36 patients developed biochemical recurrence or PSA persistence. Biochemical recurrence occurred in 11 of 344 patients (3.2%) with negative surgical margins and in 25 of 126 patients (19.8%) with positive surgical margins. The median time to event was 7.5 months. Kaplan–Meier analysis demonstrated significant differences in biochemical recurrence-free survival according to pathological stage (log-rank *p* < 0.001). At 24 months, biochemical recurrence-free survival was 93.7% for pT2 disease, 91.5% for pT3a disease, and 82.1% for pT3b disease (Figure 4).

## 4. Discussion

This study provides a comprehensive real-world analysis of a complete institutional transition from open to robot-assisted radical prostatectomy (RARP), together with contemporary perioperative and early oncological outcomes.

The transition from open radical prostatectomy (ORP) to RARP occurred rapidly and was accompanied by a more-than-fivefold increase in annual surgical volume. Beyond the adoption of robotic technology, this transition reflected a broader institutional transformation involving workflow optimization, team coordination, and standardized perioperative pathways. Such comprehensive transitions are rarely reported from single centers and provide valuable insight into the implementation of robotic surgery within a structured healthcare system.

The observed reduction in operative time and hospital stay highlights the impact of both individual and institutional learning processes. These improvements were achieved without compromising safety, as demonstrated by the low rate of major complications (0.8%). Perioperative outcomes in this cohort were favorable, with minimal blood loss, no requirement for transfusion, and reoperation. The reduction in hospital stay to predominantly 1 day reflects the successful implementation of optimized perioperative care pathways.

In addition to these global improvements, our analysis provides further insight into the learning process by demonstrating both surgeon-specific and institutional learning curves. All surgeons showed a consistent reduction in operative time, as reflected by negative regression slopes. The magnitude of improvement varied according to experience, with steeper slopes observed in surgeons earlier in their robotic practice, indicating active skill acquisition. In contrast, surgeons with higher case volumes demonstrated flatter slopes, consistent with performance stabilization. These findings are consistent with previous reports suggesting that increasing institutional experience may contribute to more consistent operative performance over time [19,20].

Early oncological outcomes were highly favorable and consistent with those reported by other high-volume centers [2,21,22]. Kaplan–Meier analysis further demonstrated favorable biochemical recurrence-free survival, particularly in patients with organ-confined disease, while patients with locally advanced pathology showed lower recurrence-free survival over time, consistent with their less favorable pathological characteristics. Biochemical recurrence occurred more frequently among patients with positive surgical margins. However, recurrence was also observed in a subset of margin-negative patients, highlighting the multifactorial nature of disease progression after radical prostatectomy.

Data regarding adjuvant or salvage radiotherapy and androgen deprivation therapy were not systematically available and were therefore not included in the analysis. The subgroup analyses according to pathological stage should be interpreted cautiously because of the relatively short follow-up period and the limited number of recurrence events, particularly within the higher-risk pathological subgroups.

Pathological findings further support the oncological relevance of the presented cohort. Although the majority of patients had organ-confined disease, more than one quarter demonstrated locally advanced pathology (pT3 disease), and a substantial proportion harbored higher-grade tumors on final pathology. In this context, the observed positive surgical margin rate should be interpreted in relation to pathological stage, tumor biology, and the nerve-sparing philosophy adopted at our institution. Whenever oncologically feasible, neurovascular bundle preservation was pursued, including in selected patients with higher-risk disease, reflecting an effort to balance oncological control with functional preservation. Importantly, despite the observed margin rates and the presence of adverse pathological features, the overall incidence of biochemical recurrence remained low during the available follow-up period. These findings suggest that positive surgical margins should not be viewed in isolation and support the overall oncological adequacy of RARP within a mature robotic program, although continued long-term follow-up remains essential.

The near-complete replacement of ORP by RARP reflects the growing adoption of robotic surgery as the predominant surgical approach for localized prostate cancer in many contemporary high-volume centers [2,23]. This shift likely reflects the perceived advantages of robotic surgery, including favorable perioperative outcomes, procedural reproducibility, and increasing scalability of robotic platforms. Our findings are consistent with outcomes reported from other high-volume European centers, confirming that robotic prostatectomy can be successfully implemented across different healthcare systems [21,24].

Our findings are also consistent with recent contemporary reports evaluating robotic prostatectomy across different robotic platforms and healthcare settings. Recent multicenter analyses have demonstrated that favorable perioperative outcomes, low complication rates, and acceptable oncological results can be achieved beyond the initial adoption phase, supporting the broader reproducibility of robotic prostatectomy within structured surgical programs [25,26]. Although the present study was not designed to compare robotic platforms, these observations further support the growing maturity and standardization of robotic prostatectomy in contemporary practice.

One of the key observations of this study is the favorable perioperative safety profile associated with robot-assisted surgery. The combination of improved visualization, instrument precision, and standardized surgical workflows contributes to reduced intraoperative blood loss, near-zero transfusion rates, and low complication rates. Furthermore, the reduction in hospital stay to predominantly 1 day reflects not only faster recovery but also decreased exposure to hospital-related complications [8,27]. These benefits were achieved consistently across a large patient cohort, suggesting that comparable outcomes can be reproduced at the institutional level within a structured robotic program [21,28].

In addition to system-level factors, the surgical technique itself may have contributed to procedural standardization and operative efficiency. Throughout the study period, a modified anterior “hood” technique with maximal preservation of the detrusor apron and avoidance of surgical clips was routinely employed. Although previous studies have suggested potential functional benefits of this approach, functional outcomes were not systematically collected in the present cohort and therefore could not be evaluated directly [15,29].

This study has several limitations. Its retrospective design and relatively short follow-up period limit the assessment of long-term oncological outcomes. In addition, functional outcomes, including urinary continence and erectile function, were not systematically collected within the institutional database and therefore could not be analyzed. As a result, the present study focuses primarily on perioperative safety, operative efficiency, pathological findings, and early oncological outcomes.

Future research should focus on long-term oncological outcomes, including biochemical recurrence and cancer-specific survival, in the context of high-volume robotic programs. In addition, standardized reporting of functional outcomes, particularly urinary continence and erectile function, will be essential to fully evaluate the benefits of specific surgical techniques such as the modified anterior “hood” approach. Further studies should also explore the relationship between surgical technique refinement, institutional learning, and patient-reported outcomes, as well as the role of emerging technologies in further improving perioperative safety.

## 5. Conclusions

Robot-assisted radical prostatectomy provides favorable perioperative safety, minimal morbidity, and favorable early oncological outcomes in a high-volume setting. The institutional transition from open to robotic prostatectomy demonstrates that large-scale adoption of robotic surgery can be achieved within a structured institutional framework. The observed surgeon-specific and institutional learning curves highlight the importance of experience, mentorship, and program development in achieving efficient and consistent outcomes. Taken together, these findings support the feasibility and safety of implementing RARP as the institutional standard within a structured robotic program.

## Figures and Tables

**Table 1:** Baseline characteristics of patients undergoing RARP (2023–2025).

Variable	Value
Age (years), median (IQR)	66 (61–71) (*n* = 520)
PSA (ng/mL), median (IQR)	7.1 (5.2–10.0) (*n* = 489)
**Biopsy ISUP Grade, *n* (%)**	
1	74 (15.2)
2	234 (48.1)
3	97 (20.0)
4	54 (11.1)
5	27 (5.6)
Total	486 (100)
**EAU Risk Group, *n* (%)**	
Low risk	61 (12.6)
Intermediate risk	314 (64.9)
High risk	109 (22.5)
Total	484 (100)

Percentages are calculated based on the available data for each variable. Differences in denominators reflect occasional missing data in routine clinical documentation. IQR, interquartile range; PSA, prostate-specific antigen; ISUP, International Society of Urological Pathologists; EAU, European Association of Urology.

**Table 2:** Surgical and perioperative outcomes of patients undergoing RARP (2023–2025).

Variable	Value
Operative time (min), median (IQR)	178 (148–210) (*n* = 520)
Estimated blood loss (mL), median (IQR)	100 (50–100) (*n* = 520)
Length of stay (days), median (IQR)	1 (1–2) (*n* = 520)
Transfusion rate, *n* (%)	0 (0) (*n* = 520)
Major complications (Clavien–Dindo ≥IIIa), *n* (%)	4 (0.8) (*n* = 520)
Full nerve-sparing (bilateral Tewari Grade 1), *n* (%)	332 (67.5) (*n* = 492)
Partial nerve-sparing, *n* (%)	158 (32.1) (*n* = 492)
Non-nerve-sparing, *n* (%)	2 (0.4) (*n* = 492)
Extended pelvic lymph node dissection (ePLND), *n* (%)	59 (11.3) (*n* = 520)
Lymph node yield, median (IQR)	10 (5–14) (*n* = 59)

Percentages are calculated based on the available data for each variable. IQR, interquartile range; ePLND, extended pelvic lymph node dissection.

**Table 3:** Pathological and early oncological outcomes of patients undergoing RARP (2023–2025). Percentages are calculated based on the available data for each variable. Differences in denominators reflect occasional missing pathological or follow-up data. Patients who did not undergo extended pelvic lymph node dissection (ePLND) were classified as pNx. Biochemical recurrence included PSA persistence, defined as failure of PSA to decline below the lower limit of assay detection following radical prostatectomy. ISUP, International Society of Urological Pathologists; ePLND, extended pelvic lymph node dissection.

Variable	Value
Pathological ISUP Grade, *n* (%)	
1	7 (1.5)
2	279 (59.9)
3	125 (26.8)
4	17 (3.7)
5	37 (8.0)
Total	465 (100)
Pathological stage, *n* (%)	
pT2	340 (72.5)
pT3a	78 (16.6)
pT3b	51 (10.9)
Total	469 (100)
Nodal status, *n* (%)	
pNx	403 (87.2)
pN0	43 (9.3)
pN1	16 (3.5)
Total	462 (100)
Surgical margin status, *n* (%)	
R0	344 (73.2)
R1	126 (26.8)
Total	470 (100)
R1 rate according to pathological stage, *n* (%)	
pT2	66 (19.4)
pT3a	32 (41.0)
pT3b	27 (52.9)
Biochemical recurrence or PSA persistence according to margin status, *n* (%)	
R0	11 (3.2)
R1	25 (19.8)

## Data Availability

The data supporting the findings of this study are not publicly available due to privacy and ethical restrictions but are available from the corresponding author upon reasonable request.

## Abbreviations

The following abbreviations are used in this manuscript:

BCR	Biochemical recurrence
IQR	Interquartile range
ISUP	International Society of Urological Pathologists
ORP	Open radical prostatectomy
PSA	Prostate-specific antigen
RARP	Robot-assisted radical prostatectomy
